# Supplementary material for: Why flying insects gather at artificial light
Source: Nat Commun. 2024 Jan 30;15:689. doi: 10.1038/s41467-024-44785-3 (PMC10827719; doi:10.1038/s41467-024-44785-3)
Supplement: Supplementary file 1 — Supplementary Information [file 41467_2024_44785_MOESM1_ESM.pdf]

Why flying insects gather at artificial light

Samuel T Fabian, Yash Sondhi, Pablo Allen, Jamie Theobald, Huai-Ti Lin

Supplementary Figures

Supplementary Figure 1

Field Stereo-Videography

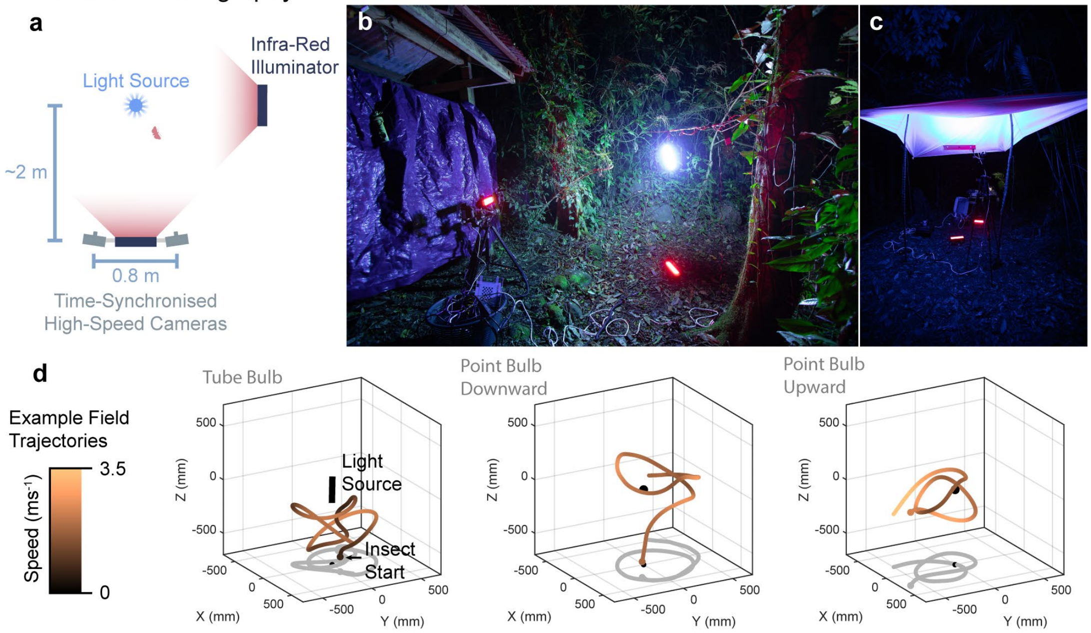

Supplementary Figure 1: a, A diagram of the high-speed stereo-videographic field setup from a top-down perspective. **b,** A photograph of the field setup at CIEE, Montevideo, Costa Rica. **c,** A photograph of the diffuse canopy experimental setup. **d,** Example digitised 3D flight trajectories from the field.

Supplementary Figure 2

Laboratory Motion Capture

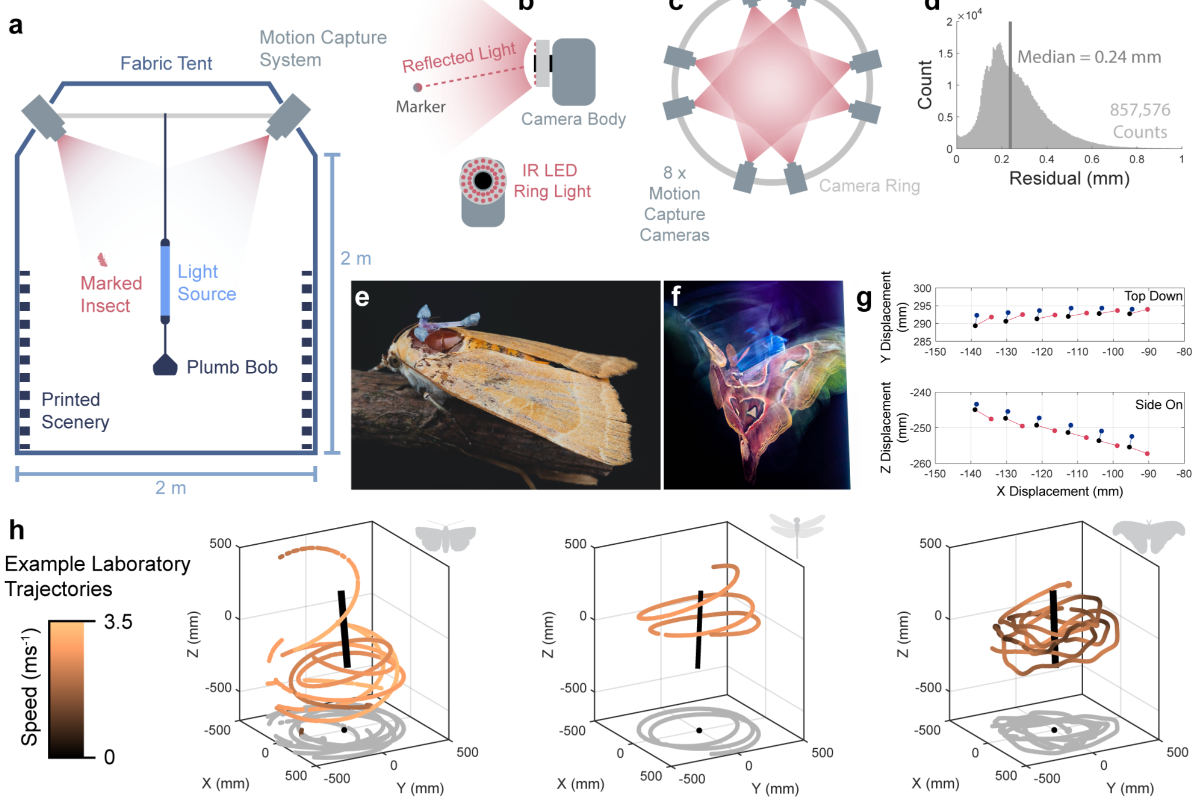

Supplementary Figure 2: a, A diagrammatic representation of the setup for laboratory-based motion capture experiments. **b,** A diagram of the principles underlying motion capture recording. **c,** A diagram of the orientation of the motion capture recordings from a top-down perspective. **d,** A histogram of the residual (distance between lines of sight for multiple cameras, reflecting estimated error) across all marker recordings. **e,** *Noctua fimbriata* with marker-frame attached to the dorsal side of the thorax. **f,** *Attacus lorquinii* in flight with marker-frame attached to the dorsal side of the thorax. **g,** Example trace of 6 successive frames of reconstructed markers from an insect in flight. **h,** Example 3D tracks for 3 of the study species: *Noctua sp.* (left), *Sympetrum striolatum* (middle), *Attacus lorquinii* (right). Source data are provided as a Source Data file.

Supplementary Figure 3

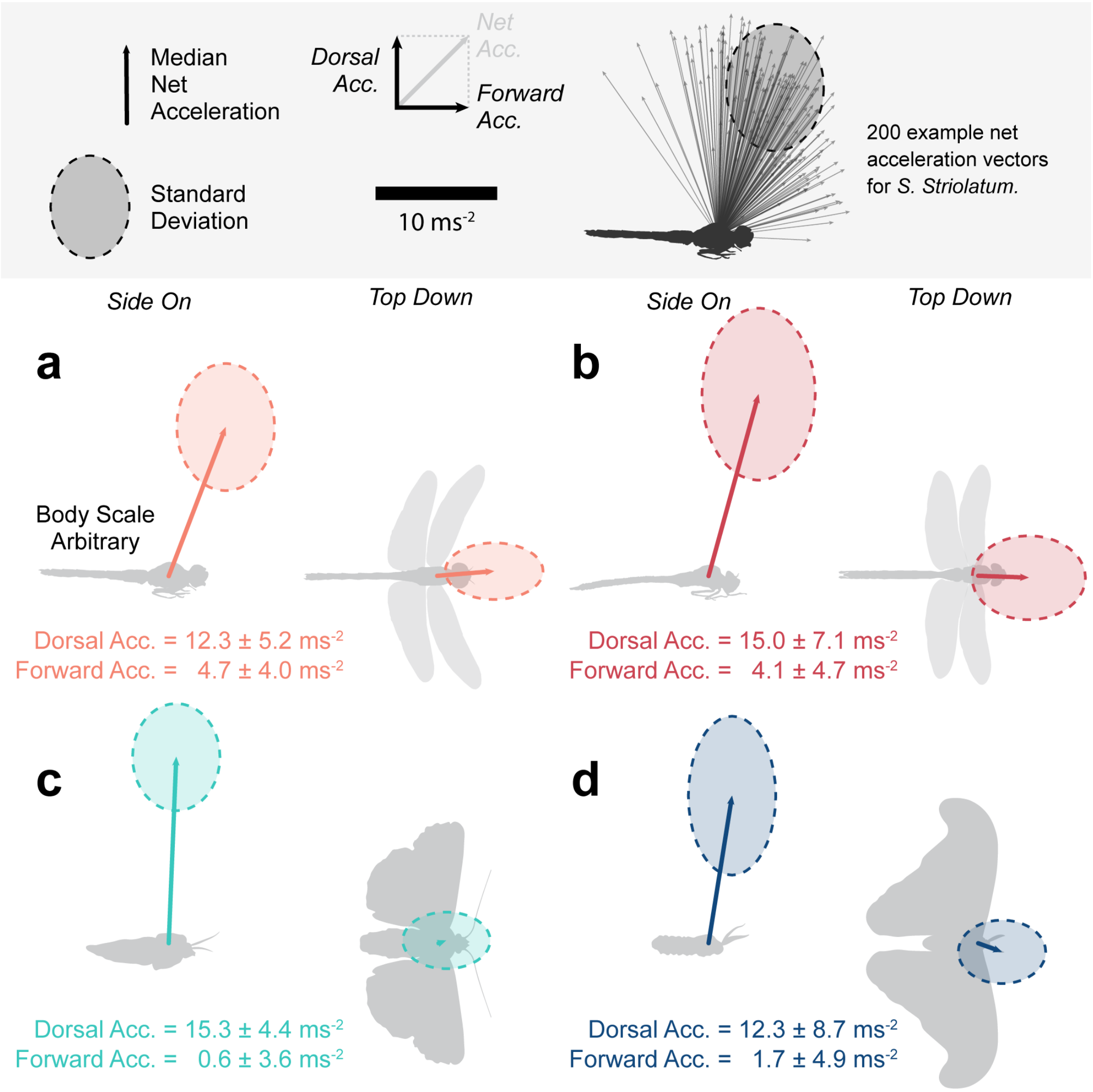

Supplementary Figure 3: Our flight simulations assumed that insects created accelerations within a limited range of directions relative to their bodies. We measured the body-centric acceleration of insects flying within our motion capture arena, accounting for the component counteracting gravity. The mean and standard deviation of net accelerations (excluding gravitational acceleration) during flight are plotted in the body reference frame for a single individual of **a,** *Sympetrum striolatum*, **b,** *Aeshna mixta*, **c,** *Noctua sp.*, and **d,** *Attacus lorquinii*. Acceleration vectors were averaged per wingbeat to account for within wingbeat repeated variation for each species before being included in the dataset. Source data are provided as a Source Data file.

Supplementary Figure 4

Celestial Compass Simulations

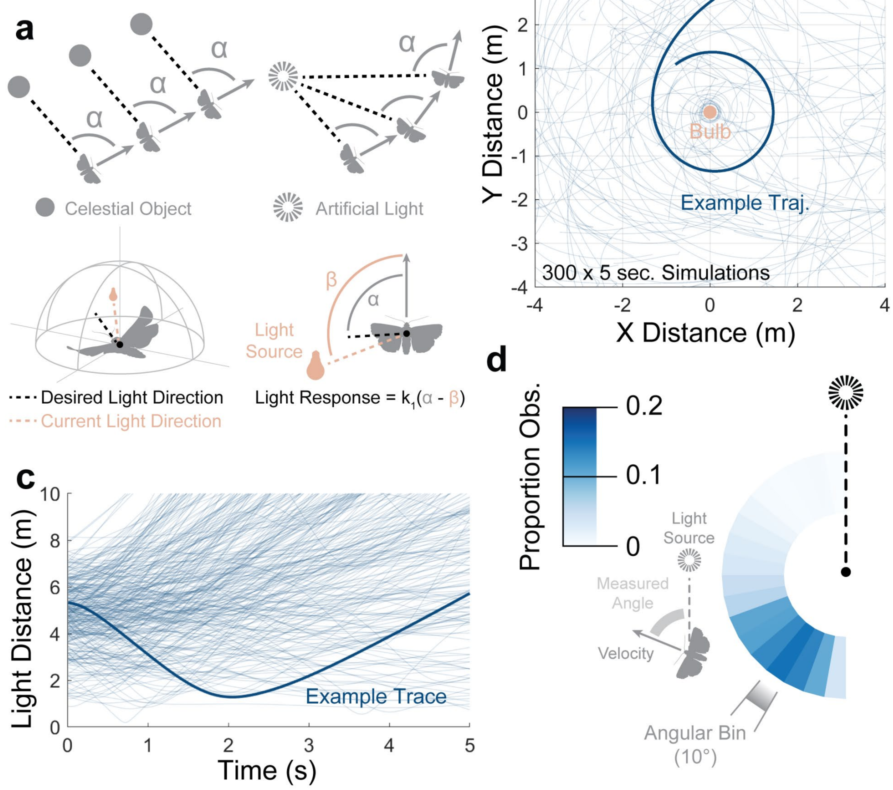

Supplementary Figure 4: One suggested hypothesis for insect light entrapment involves the confusion of a celestial compass cue. **a,** (*Top Left*) Flying insects can use distant celestial objects as compass cues to maintain a consistent heading. (*Top Right*) If they confused an artificial light source for their compass cue, its proximity would lead to their travelling in curving spirals. (*Bottom row*) We adapted our flight simulations such that agents would attempt to keep the light source close to an arbitrary but fixed visual location (set by the initial line-of-sight to the light). Agents steered in proportion to the magnitude of the discrepancy between the desired and current light directions. **b,** Top-down plots of the trajectories taken by 300, 5-second, simulations with randomised free parameters. **c,** Overlaid trajectories of the simulations' distance to light over time show a trend to move further from the light source. Agents travelling with a confused celestial compass cue did not display the tendency to travel orthogonally to the light source seen in real insects and in DLR simulations. **d,** The orientation of the velocity vectors of the celestial compass simulations relative to the light source, coloured by the proportion observed. Source data are provided as a Source Data file.

Supplementary Figure 5

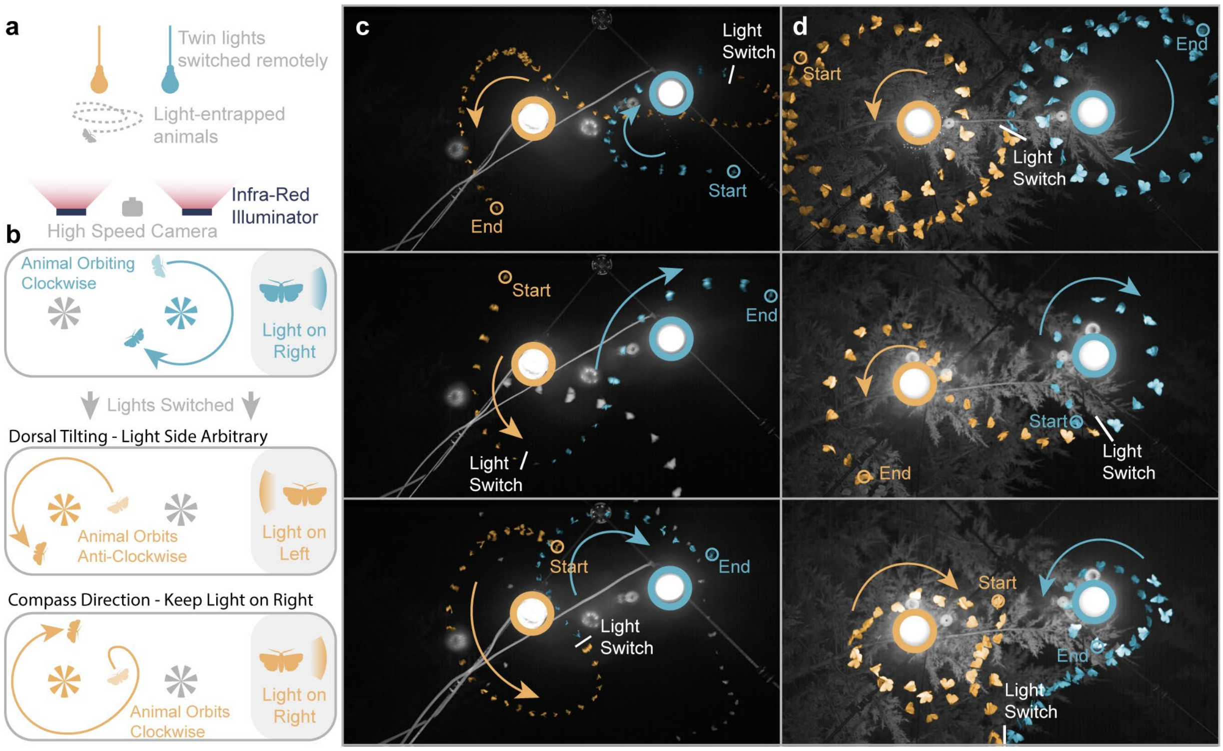

Supplementary Figure 5: If insects orbiting light sources were confusing the light with a compass cue such as the moon, we would expect them to maintain an orbit only in one direction. We switched between two lights when an insect began orbiting, to test whether they would maintain the direction of their orbit on a new light. Throughout, colour indicates which light is currently on. **a,** A diagram of the light switching set-up. **b,** Illustrations of the alternative path outcomes from light switching, given an initial insect travelling in an anti-clockwise direction. Orbiting moths readily switched their orbiting when the lights were changed. Image overlays (every 20 ms) of the light switching with nocturnal **c,** and diurnal **d**, species viewed from below, with insects false-coloured corresponding to the light concurrently lit. Arrows indicate direction of travel.

Supplementary Figure 6

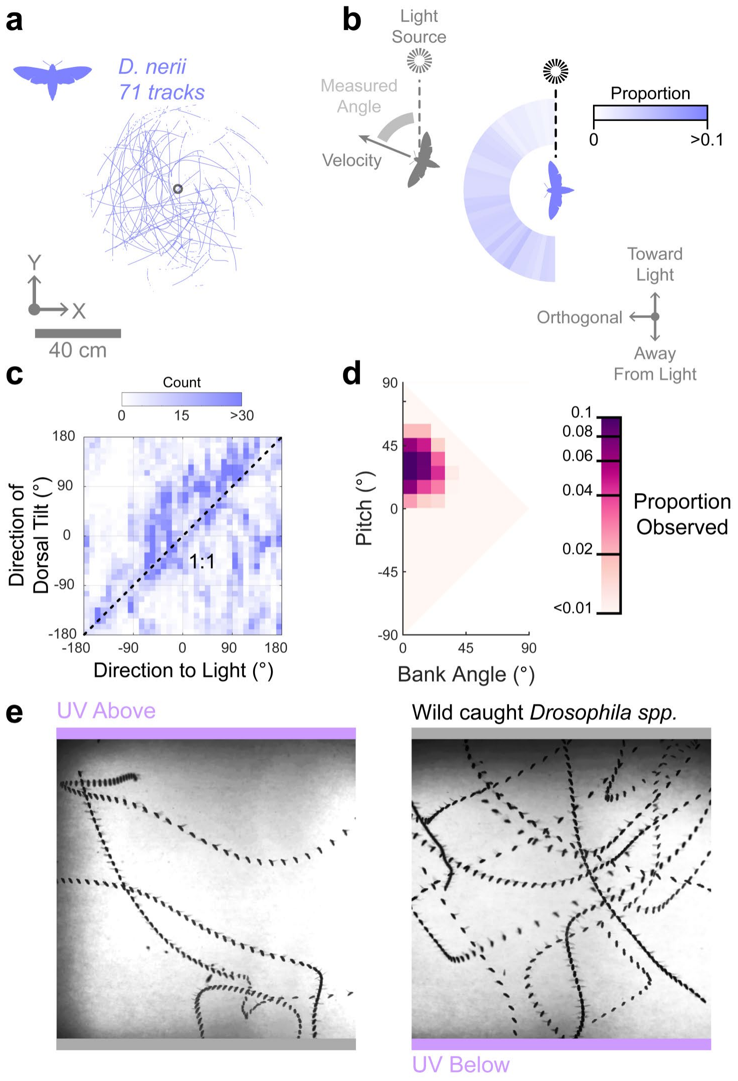

Supplementary Figure 6: Two species did not display light-centric behavioural motifs in the laboratory environment. **a,** Top-down plotted flight tracks for the Oleander Hawkmoth (*Daphnis nerii*). **b,** The relative prevalence of the insects' horizontal velocity orientation relative to the light. **c,** The directions of the vectors of the insect's dorsal axis, and connecting the insect to the light source are compared on the ground plane. Horizontal reference frame is fixed but arbitrary. **d,** The orientations of our measured insects are plotted on axes of pitch and bank angle. **e,** Wild caught *Drosophila spp*. fly under (*left*) and over (*right*) a UV LED bulb. Source data are provided as a Source Data file.

Supplementary Figure 7

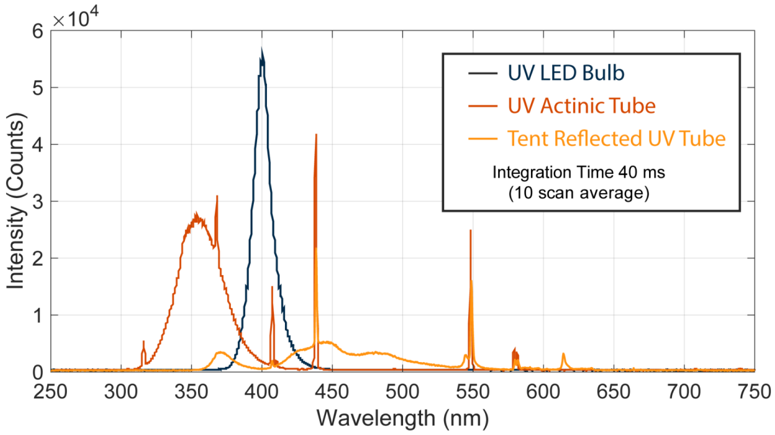

Supplementary Figure 7: The emittance spectrum of the two UV light types used in experiments, and the reflectance spectrum of the laboratory behaviour tent. Source data are provided as a Source Data file.

Supplementary Figure 8

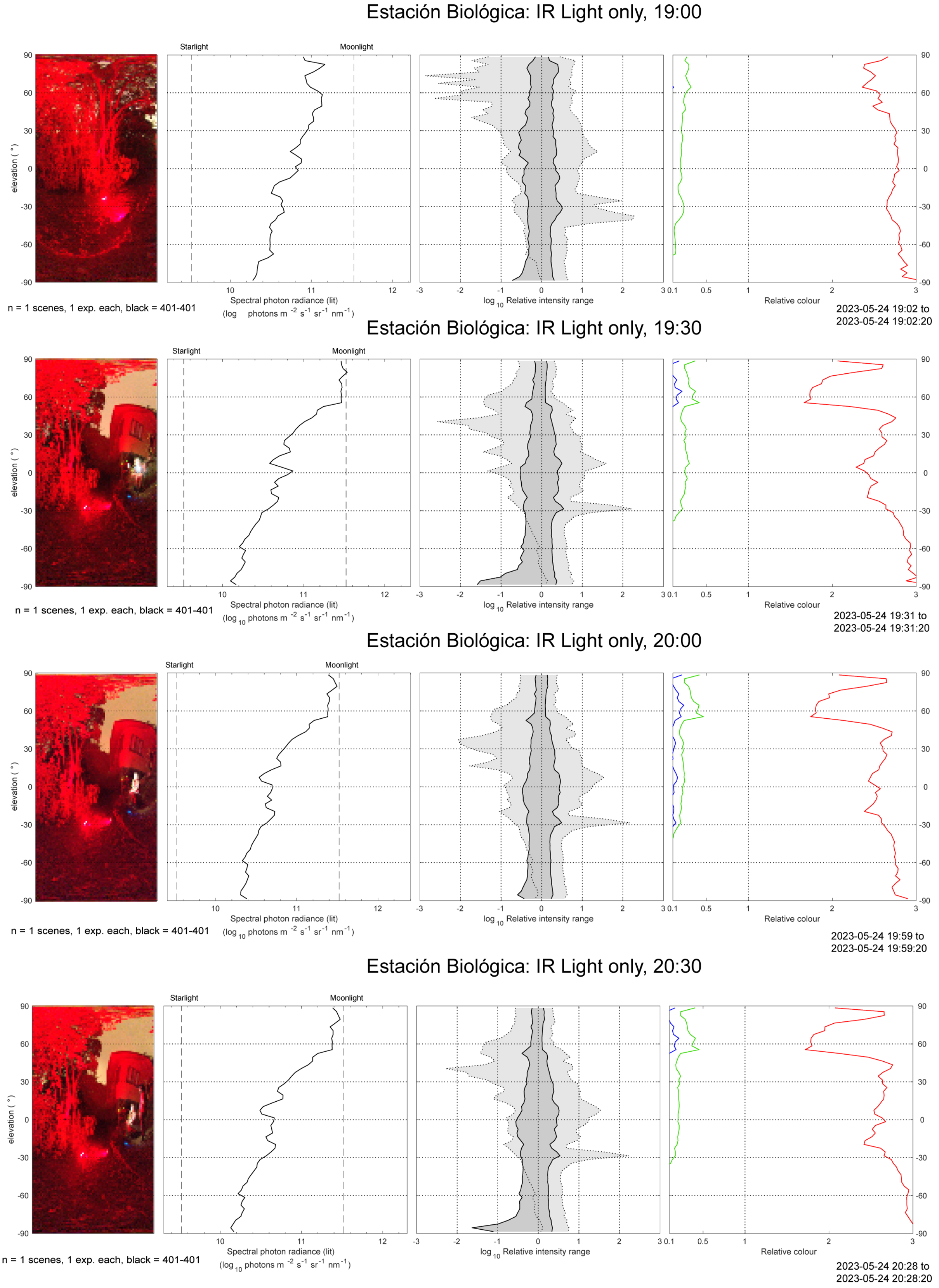

Supplementary Figure 8: Environmental light field measurements at the Estación Biológica Field site, Monteverde, Costa Rica. Source data are provided as a Source Data file.
