# Supplementary material for: Why flying insects gather at artificial light
Source: Nat Commun. 2024 Jan 30;15:689. doi: 10.1038/s41467-024-44785-3 (PMC10827719; doi:10.1038/s41467-024-44785-3)
Supplement: Supplementary file 3 — Description of Additional Supplementary Files [file 41467_2024_44785_MOESM3_ESM.pdf]

Description of Additional Supplementary Files

File Name: Supplementary Data 1
Description: Metadata for high-speed stereographic field videos including information on calibration and plumbline video names associated with each set of synchronised videos, location, treatment, and putative identifications of insect order or genus.

File Name: Supplementary Data 2
Description: Summary of video data including the duration and variability of video clips under different treatment conditions.

File Name: Supplementary Data 3
Description: Metadata for motion capture trajectories used in the study, including information on the marker being tracked, settings, lighting conditions, and specific details about the duration and frame rate of each clip.

File Name: Supplementary Data 4
Description: Summary of motion capture data including the duration and variability of tracks under different treatment conditions.

File Name: Supplementary Data 5
Description: Metadata and summary statistics for the 3D digitised trajectories, IDs refer to videos from Supplementary Data 3, and video files are available within the repository.

File Name: Supplementary Movie 1
Description: Nocturnal flying insects demonstrating an orbiting flight motif around an artificial light source. Recorded in Monteverde, Costa Rica.

File Name: Supplementary Movie 2
Description: Nocturnal flying insects demonstrating a stalling flight motif around an artificial light source. Recorded in Monteverde, Costa Rica.

File Name: Supplementary Movie 3
Description: Nocturnal flying insects demonstrating an inverting flight motif around an artificial light source. Recorded in Monteverde, Costa Rica.

File Name: Supplementary Movie 4
Description: Insects (*Noctua sp*., *Attacus lorquinii*, and *Sympetrum striolatum*) flying around artificial light sources within the laboratory environment. Recorded at Imperial College London.

File Name: Supplementary Movie 5
Description: Different small insect taxa flying in a small acrylic box with UV (400 nm) light coming from either above, or below. Recorded at Imperial College London.

File Name: Supplementary Movie 6
Description: Released Pieridae butterflies orbiting beneath UV (400 nm) LED bulbs change their direction of orbit when switching to a new bulb. Recorded in Cambridge, UK.

File Name: Supplementary Movie 7
Description: Oleander hawkmoths (*Daphnis nerii*) flying near an upward facing UV (400 nm) LED bulb in the laboratory environment. Recorded at Imperial College London.
