# Supplementary material for: Why flying insects gather at artificial light
Source: Nat Commun. 2024 Jan 30;15:689. doi: 10.1038/s41467-024-44785-3 (PMC10827719; doi:10.1038/s41467-024-44785-3)
Supplement: Supplementary file 16 — Reporting Summary [file 41467_2024_44785_MOESM16_ESM.pdf]

nature portfolio

Corresponding author(s): Samuel T. Fabian & Yash Sondhi

Last updated by author(s): Nov 30, 2023

# Reporting Summary

Nature Portfolio wishes to improve the reproducibility of the work that we publish. This form provides structure for consistency and transparency in reporting. For further information on Nature Portfolio policies, see our Editorial Policies and the Editorial Policy Checklist.

## Statistics

For all statistical analyses, confirm that the following items are present in the figure legend, table legend, main text, or Methods section.

| n/a | Confirmed | |
|---|---|---|
| ☐ | ☒ | The exact sample size (*n*) for each experimental group/condition, given as a discrete number and unit of measurement |
| ☐ | ☒ | A statement on whether measurements were taken from distinct samples or whether the same sample was measured repeatedly |
| ☐ | ☒ | The statistical test(s) used AND whether they are one- or two-sided<br>*Only common tests should be described solely by name; describe more complex techniques in the Methods section.* |
| ☒ | ☐ | A description of all covariates tested |
| ☐ | ☒ | A description of any assumptions or corrections, such as tests of normality and adjustment for multiple comparisons |
| ☐ | ☒ | A full description of the statistical parameters including central tendency (e.g. means) or other basic estimates (e.g. regression coefficient) AND variation (e.g. standard deviation) or associated estimates of uncertainty (e.g. confidence intervals) |
| ☐ | ☒ | For null hypothesis testing, the test statistic (e.g. *F*, *t*, *r*) with confidence intervals, effect sizes, degrees of freedom and *P* value noted<br>*Give P values as exact values whenever suitable.* |
| ☒ | ☐ | For Bayesian analysis, information on the choice of priors and Markov chain Monte Carlo settings |
| ☒ | ☐ | For hierarchical and complex designs, identification of the appropriate level for tests and full reporting of outcomes |
| ☒ | ☐ | Estimates of effect sizes (e.g. Cohen's *d*, Pearson's *r*), indicating how they were calculated |

*Our web collection on statistics for biologists contains articles on many of the points above.*

## Software and code

Policy information about availability of computer code

| | |
|---|---|
| Data collection | High-speed video recordings in the field were made with FasMotion 2.5.3 (fastec). Motion capture recordings in the laboratory were made with Qualisys Track Manager 2021 (Qualisys). Light measurements were made with OceanView 2.0 and open-source ELF-software (https://github.com/sciencedjinn/elf). |
| Data analysis | Data analysis was performed using MATLAB 2021a and python using custom scripts. Example scripts have been provided with the supporting data made available with this article (https://doi.org/10.6084/m9.figshare.24771978). |

For manuscripts utilizing custom algorithms or software that are central to the research but not yet described in published literature, software must be made available to editors and reviewers. We strongly encourage code deposition in a community repository (e.g. GitHub). See the Nature Portfolio guidelines for submitting code & software for further information.

## Data

Policy information about availability of data

All manuscripts must include a data availability statement. This statement should provide the following information, where applicable:

- Accession codes, unique identifiers, or web links for publicly available datasets
- A description of any restrictions on data availability
- For clinical datasets or third party data, please ensure that the statement adheres to our policy

The raw stereo-videos and processed trajectories associated with this manuscript are available via Figshare (https://doi.org/10.6084/m9.figshare.24771978). Example high-speed videos of our experiments are provided in Supplementary Videos 1-7. These data include both the video-tracked 3D trajectories and 6-DoF

laboratory motion capture trajectories of insects around light. All processed data used to make figure panels is available in a source data file. The source data file also contains the all the required data to replicate our statistical testing of hypotheses.

## Research involving human participants, their data, or biological material

Policy information about studies with human participants or human data. See also policy information about sex, gender (identity/presentation), and sexual orientation and race, ethnicity and racism.

| | |
|---|---|
| Reporting on sex and gender | Not applicable |
| Reporting on race, ethnicity, or other socially relevant groupings | Not applicable |
| Population characteristics | Not applicable |
| Recruitment | Not applicable |
| Ethics oversight | Not applicable |

Note that full information on the approval of the study protocol must also be provided in the manuscript.

# Field-specific reporting

Please select the one below that is the best fit for your research. If you are not sure, read the appropriate sections before making your selection.

☐ Life sciences ☐ Behavioural & social sciences ☒ Ecological, evolutionary & environmental sciences

For a reference copy of the document with all sections, see nature.com/documents/nr-reporting-summary-flat.pdf

# Ecological, evolutionary & environmental sciences study design

All studies must disclose on these points even when the disclosure is negative.

| | |
|---|---|
| Study description | Our study recorded the flight behaviour of night-flying insects around artificial lights. We used stereo high-frame-rate stereo videography in the field and infrared motion capture within the laboratory to reconstruct the trajectory and orientation of insects in free flight. We were able to reconstruct 345 trajectories from the field, and 599 trajectories from the lab. Exact treatment and species breakdowns are given within Tables 1 & 3 of the manuscript. |
| Research sample | Our experiments were designed to test a large sample of flying insects represented at nocturnal light traps. We also included diurnal species to test for whether measured effects were specific to nocturnal species. Our sample encompassed species with greatly differing body lengths (3mm to 7 cm) and different flight styles. Within the field, we were unable to distinguish the exact insect taxa filmed with certainty. A majority of field trajectories came from night-flying moths (Lepidoptera), we identified the insects to order where possible and provide this metadata with the videos (Supplemental Data 1). For a small fraction of insects, we identified them to the genus or species where possible, by capturing and photographing them prior to their use in experiments (Supplemental Data 1). The identifications were made using photographs of live animals which were eventually released and therefore should be treated as tentative. Within laboratory motion-capture experiments, we used adults of 5 species (Sympetrum striolatum (n = 12), Aeshna mixta (n = 2), Noctua sp. (n = 10), Attacus lorquinii (n = 3), and Daphnis nerii (n = 3). The first two represented day-flying insects not normally represented in light-trapping data. The latter three represented night-flying insects known to collect around artificial light. We also used the European honeybee Apis mellifera (n = 6), wild-caught Drosophila spp. (n = 21), other Diptera (n = 31), and other small insects (See Table 4) for a coarse assessment of reactions to light-source direction in smaller flying insects, since we could not easily attach motion-capture markers onto them. Light switching experiments were conducted with wild insects, largely moths, butterflies and wasps (See Table 5) for a breakdown. |
| Sampling strategy | We did not choose a predefined sample size, but tried to equalize the number of samples for each condition. We obtained a minimum 50 recordings per field condition, the upper limit being constrained by the duration of the field trip. Our rationale for deciding against further sampling was the recovery of robust pattern within each experimental treatment. To confirm the identity of the various insect orders flying to light, we caught and identified different orders at a light sheet and after allowing them to dark adapt, released them close to the light in a smaller indoor chamber. Similarly, for our laboratory motion capture experiments, data collection was primarily constrained by number of animals available and the duration of flight bouts they displayed. Finding highly robust trends after analyzing the data between groups, we surmised our sample sizes were sufficient. |
| Data collection | Field data was recorded by YS, SF and JT with the assistance of PA using high-speed cameras and infrared lights and different light sources. For most videos, we recorded insects as they appeared in the field of view and we could capture in the camera buffer. For the smaller fraction of the videos to identify orders, we captured insects 2-3 hours before the trial at a UV LED light and were released after they were dark adapted and their trajectories were recorded by the two camera setup.<br>Insects were tagged and flown in the laboratory motion capture arena by SF. Footage of flies, bees and moths in the light switching experiments was collected by SF. |
| Timing and spatial scale | Field data was collected from sites in Costa Rica: Estación Biológica Monteverde and CIEE field station from January 28th to February 8th, 2022. A subsequent round of data collection took place from May 20th to 24th, 2023, at these same sites.<br>Laboratory data was collected at Dr. Hua-Ti Lin's laboratory in Imperial College London in 2021 and 2022. Light switching field |

| | |
|---|---|
| | experiments were conduced in Cambridge, UK in summer 2022 and 2023. |
| Data exclusions | For field data, we include all the data collected and provide them as supplementary videos, however, trajectories were analyzed for videos where the tracking software was able to provide a successful 3D path, cases where there were errors in trajectory reconstruction and digitsation are mentioned in the metadata. Flight data from motion-capture were excluded below 0.4 m/s. We excluded these points to avoid including insects that had landed within the arena and were walking rather than flying. Our motion-capture did not provide video feedback, preventing us from determining this visually. |
| Reproducibility | Many of our findings are readily reproducible without specialist equipment. A large proportion of flying wild-insects are influenced by artificial light, and some of the behavioural motifs we describe are visible to the naked eye, once described. We also examine the videos annotating presence or absence of these motifs and provide summaries of the same. Our findings were highly consistent between different individuals (30 individual insects in motion capture) and different insect taxa (accross 10 different insect orders). Findings observed under laboratory conditions matched observations of wild insects recorded in the field. |
| Randomization | As per the nature of this study, we did not allocate research organisms into groups for specific treatments. Within our laboratory environment, we attempted to record each species under all the lighting conditions (though this was not always possible due to constraints created by the timing of emergence). Within the field, we did not control the arrival of insects at light-sources, effectively randomising participant species (we recorded at the same site for multiple nights with all lighting conditions). |
| Blinding | Blinding was not possible in this study as the light treatments provided are highly salient to the experimenter. |

Did the study involve field work? ☒ Yes ☐ No

## Field work, collection and transport

| | |
|---|---|
| Field conditions | The conditions over 28th Jan -7th Feb 2022 varied between 16C-23 C at night and humidity between 70-85 %. Wind was much less at CIEE (<0.1 m/s) and temperatures were higher (20-23 C), but at Monteverde, the site was more exposed to the wind (0-1.5m/s) and rain. We had light rain on 30 and 31st Jan 2022 with moderate wind, but minimal rain and lower winds on other days where we conducted field recordings. For 20th to 22nd May 2023 in CIEE, temperature was not variable during the recording time 21-23 C with relative humidity between 71 %and 86 %. For 23-24th May at CIEE, it ranged from 18-20 C and 82-87% relative humidity. The light levels are also provided in the paper. |
| Location | Field recordings were made at the Estacion Biologica, Monteverde, Costa Rica (10.3190, -84.8085) and CIEE(10.2819, -84.7955), Monteverde, Costa Rica |
| Access & import/export | Our recordings were made under permit numbers M-P-SINAC-PNI-ACAT-024-2020 and R-SINAC-ACG-PI-016-2022 issued by SINAC (National System of Conservation Areas). We did not capture or kill insects flying near the lights, nor did we take physical samples. In some cases for the purpose of identification, they were caught photographed and released after the experiments. |
| Disturbance | Our light treatments created a small amount of localised light pollution for a period of up to 4 hours during recording sessions. The effected area was similar to a single standard light trap used to catch insects. Insects were neither captured nor killed, leaving minimal long-term disturbance to the area in which recordings were made. |

# Reporting for specific materials, systems and methods

We require information from authors about some types of materials, experimental systems and methods used in many studies. Here, indicate whether each material, system or method listed is relevant to your study. If you are not sure if a list item applies to your research, read the appropriate section before selecting a response.

### Materials & experimental systems

| n/a | Involved in the study |
|---|---|
| ☒ | ☐ Antibodies |
| ☒ | ☐ Eukaryotic cell lines |
| ☒ | ☐ Palaeontology and archaeology |
| ☐ | ☒ Animals and other organisms |
| ☒ | ☐ Clinical data |
| ☒ | ☐ Dual use research of concern |
| ☒ | ☐ Plants |

### Methods

| n/a | Involved in the study |
|---|---|
| ☒ | ☐ ChIP-seq |
| ☒ | ☐ Flow cytometry |
| ☒ | ☐ MRI-based neuroimaging |

## Animals and other research organisms

Policy information about studies involving animals; ARRIVE guidelines recommended for reporting animal research, and Sex and Gender in Research

| | |
|---|---|
| Laboratory animals | This study did not use animals that have been reared in a laboratory for multiple generations. |

| | |
|---|---|
| Wild animals | For wild animals used in the high speed video recordings, we filmed them as they flew by the light and they were not captured or manipulated in any way. For high-speed recordings filmed indoors, we caught and photographed the animals in mesh cages at a light. After dark adaptation near the light source, they were released once all experiments for that night were completed. Taxonomic identifications are provided in Supplemental Data 1, but are generally only to order-level.<br>We also used wild caught (UK) small insects (total n = 80, see Table 4) for a coarse assay on reactions to the direction of light-sources in smaller flying insects than those used for motion-capture. Subjects were released after our experiments |
| Reporting on sex | Sex was not considered in the study. |
| Field-collected samples | We used adults of 5 species (Sympetrum striolatum (n=12), Aeshna mixta (n=2), Noctua sp. (n=10), Attacus lorquinii (n = 3), and Daphnis nerii (n = 3)). S. striolatum, A. mixta, and Noctua sp. were collected from the wild (either in London, UK, or Cambridge, UK). A. lorquinii and D. nerii were purchased as pupae from a supplier within the UK. All captive insects were housed in a specially constructed tent, featuring high-intensity broad-spectrum lighting on a 16:8 Light:Dark cycle. Humidity was maintained at 60-70% using custom instrumentation, and the temperature between 22 - 28°C. After use in recordings, the retroreflective marker frames were removed from all animals. Species native to the UK were released back into the wild. Species non-native to the UK remained in the tent for the remainder of their lives. Wild caught small insects (total n = 80, see Table 4) were used for a coarse assay on reactions to the direction of light-sources in smaller flying insects. They were kept for a short (<24 hrs) duration before being released. In Costa Rica, animals were mostly filmed as they flew near the light. To sample a known set of insects, for two nights, insects were caught and photographed in mesh cages at the light screen, dark adapted and then released in a large room with the light source. For light switching experiments, animals were either not captured and filmed as they came to light and in the case of diurnal animals, they were captured and released at the light. All of these animals were native to the UK and free to leave the experimental setup. |
| Ethics oversight | No ethical approval was necessary for our research subjects. |

Note that full information on the approval of the study protocol must also be provided in the manuscript.

## Plants

| | |
|---|---|
| Seed stocks | *Report on the source of all seed stocks or other plant material used. If applicable, state the seed stock centre and catalogue number. If plant specimens were collected from the field, describe the collection location, date and sampling procedures.* |
| Novel plant genotypes | *Describe the methods by which all novel plant genotypes were produced. This includes those generated by transgenic approaches, gene editing, chemical/radiation-based mutagenesis and hybridization. For transgenic lines, describe the transformation method, the number of independent lines analyzed and the generation upon which experiments were performed. For gene-edited lines, describe the editor used, the endogenous sequence targeted for editing, the targeting guide RNA sequence (if applicable) and how the editor was applied.* |
| Authentication | *Describe any authentication procedures for each seed stock used or novel genotype generated. Describe any experiments used to assess the effect of a mutation and, where applicable, how potential secondary effects (e.g. second site T-DNA insertions, mosiacism, off-target gene editing) were examined.* |
